# Blues in the Brain and Beyond: Molecular Bases of Major Depressive Disorder and Relative Pharmacological and Non-Pharmacological Treatments

**DOI:** 10.3390/genes11091089

**Published:** 2020-09-18

**Authors:** Elisabetta Maffioletti, Alessandra Minelli, Daniela Tardito, Massimo Gennarelli

**Affiliations:** 1 Genetics Unit, IRCCS Istituto Centro San Giovanni di Dio Fatebenefratelli, 25125 Brescia, Italy; alessandra.minelli@unibs.it (A.M.); massimo.gennarelli@fatebenefratelli.eu (M.G.); 2 Division of Biology and Genetics, Department of Molecular and Translational Medicine, University of Brescia, 25123 Brescia, Italy; 3 Psychiatric Unit, IRCCS Istituto Centro San Giovanni di Dio Fatebenefratelli, 25125 Brescia, Italy; daniela.tardito@uniecampus.it; 4 Department of Technical and Applied Sciences, eCampus University, 22060 Novedrate (CO), Italy

**Keywords:** major depressive disorder, antidepressant drugs, non-pharmacological treatments, electroconvulsive therapy, monoamines, serotonin, neurotrophins, brain-derived neurotrophic factor, inflammation

## Abstract

Despite the extensive research conducted in recent decades, the molecular mechanisms underlying major depressive disorder (MDD) and relative evidence-based treatments remain unclear. Various hypotheses have been successively proposed, involving different biological systems. This narrative review aims to critically illustrate the main pathogenic hypotheses of MDD, ranging from the historical ones based on the monoaminergic and neurotrophic theories, through the subsequent neurodevelopmental, glutamatergic, GABAergic, inflammatory/immune and endocrine explanations, until the most recent evidence postulating a role for fatty acids and the gut microbiota. Moreover, the molecular effects of established both pharmacological and non-pharmacological approaches for MDD are also reviewed. Overall, the existing literature indicates that the molecular mechanisms described in the context of these different hypotheses, rather than representing alternative ones to each other, are likely to contribute together, often with reciprocal interactions, to the development of MDD and to the effectiveness of treatments, and points at the need for further research efforts in this field.

## 1. Introduction

Major depressive disorder (MDD) is a disabling psychiatric condition leading to a persistent feeling of sadness and loss of interest, and it is among the top five leading causes of disability and disease burden throughout the world. MDD and other forms of clinical depression are characterized by persistent low mood with associated changes in behaviour, cognition, sleep and appetite, impaired social and occupational functioning, increased risk of self-harm or suicide, and increased mortality due to co-occurring general medical disorders [1].

The course of MDD is quite variable; indeed, many patients never, or only rarely, reach remission, considered as a period of at least two months without symptoms, and experience relapses. Moreover, although MDD may occur only once during life, people typically have multiple depressive episodes; in this case, the disease is defined as recurrent depression. For the definition of recurrence, there must be an interval of at least two consecutive months between separate episodes in which the criteria for a major depressive episode are not met. Recurrence rates are over 85% within a decade from an index depressive episode, and average approximately 50% or more within six months of apparent clinical remission if the initially effective treatment is not continued [2,3]. In turn, the chronicity of the pathology reduces drastically the probability of a complete resolution of the symptomatology.

Several international guidelines for the acute treatment of moderate to severe MDD recommend a first-line treatment with antidepressant drugs (ADs), including selective serotonin (SSRIs) and serotonin-norepinephrine reuptake inhibitors (SNRIs), older tricyclic antidepressants (TCAs) and a growing number of other types of drugs. However, although the most widespread therapeutic approach for MDD is represented by drug treatments, these are often ineffective. The most important pharmacological trials carried out on MDD patients revealed that only about one third of them respond to the first treatment with ADs [4,5]. Most patients with MDD need different pharmacological treatments, often in combination, to achieve remission of symptoms, and a high percentage of them, about 30–40%, is classified as suffering from treatment-resistant depression (TRD) [6,7]. Moreover, acute treatment with antidepressant medications has not been associated with the prevention of recurrence or relapse, but continuing or maintaining antidepressant medication regimens for patients has been associated with forestalling symptom return [8,9]. 

The inadequacy of the response to pharmacological therapies encouraged the development and the interest in non-pharmacological treatments, mainly used as adjuvants to drug treatments. Currently, several non-pharmacological treatments for MDD are available, such as electroconvulsive therapy (ECT) and cognitive behavioural therapy (CBT), which are relatively well established as having at least moderate efficacy for the short-term treatment of acute MDD [2,9,10]. A relevant issue concerning non-pharmacological treatments is that this term refers to a myriad of procedures that have in common the non-use of drugs, but which are strongly different one to each other. To date, the non-pharmacological treatments widespread in clinical practice for the treatment of MDD (particularly for severe and recurrent forms) that have demonstrated strong efficacy and have been reported in the National Institute for Health and Care Excellence guidelines update in 2019 are ECT, repetitive transcranial magnetic stimulation (rTMS) and vagus nerve stimulation (VNS) among neuromodulation strategies, and CBT and interpersonal psychotherapy (IPT) among psychotherapies [11,12,13,14].

Several molecular studies were carried out concerning the pathogenesis of MDD, but the implicated molecular mechanisms remain largely unclear, although various hypotheses have been proposed. Furthermore, studies conducted on biomarkers of MDD treatments did not allow drawing exhaustive conclusions about the mechanisms involved and the possibility to discriminate responders and non-responders; consequently, personalized medicine for MDD is far from being used in daily clinical practice.

On these bases, the present narrative review aims to perform a critical depiction of the existing research literature concerning the molecular mechanisms underlying MDD and the relative main treatments, both pharmacological and non-pharmacological.

## 2. Hypotheses about the Molecular Bases of Major Depressive Disorder 

### 2.1. Classical Hypotheses: The Monoaminergic and Neurotrophic Hypotheses

Starting from the 1950s, different theories have been proposed about the molecular mechanisms involved in MDD. The first one is founded on the so-called **monoaminergic hypothesis** and originates from the research conducted by the British chemist Bernard Brodie who, in the 1950s, was studying the effects of reserpine. Reserpine is an alcaloid extracted from the root of the climbing shrub *Rauwolfia serpentina*, which was used at the time as an anti-hypertensive drug with the disadvantage of inducing a depressive syndrome as a side effect. This substance had also been observed to induce sedation and motor slowing in rats, effects that were similar to those observed in humans; as in patients treated with reserpine, these symptoms ceased when the administration of the drug was suspended. In the brain of rats treated with resperpine, Brodie detected a strong reduction in the levels of the neurotransmitter serotonin (5-idroxytriptamine, 5-HT), whereas the concentration of 5-hydroxyindoleacetic acid, a serotonin metabolite, was elevated in the urine, indicating a degradation of the neurotransmitter [15,16].

In the following years the Swedish scientist Arvid Carlsson discovered that, besides decreasing serotonin levels, reserpine also reduced the amount of two other biogenic amines or monoamines, i.e., noradrenaline (norepinephrine) and dopamine, by interfering with their storage in synaptic vesicles and inducing a reduction in the quantities available for release into the synaptic cleft and subsequent binding to postsynaptic receptors. These findings led to the formulation of the hypothesis that serotonin, noradrenaline and dopamine are involved in the control of mood and affective/emotional functions, and that a decrease in their concentrations is the basis for the development of depressive pathology [17]. 

Further evidence in support of this hypothesis came from the discovery, in which the American scientist Nathan Kline was involved [18], that iproniazide, an antibacterial agent used to treat tuberculosis, caused an improvement in mood in patients with depressive symptoms. It was later observed that this drug is able to inhibit the mitochondrial enzyme monoamine oxidase (MAO), responsible for the degradation of the monoamines in free form located at presynaptic terminals. Through the inhibition of MAOs, iproniazide raises the presynaptic levels of monoamines, in particular serotonin and noradrenaline. This drug later proved its effectiveness in the treatment of depressed patients without tuberculosis, and other drugs with the function of inhibiting the MAOs, therefore called MAO inhibitors (MAOIs), were developed for the treatment of MDD [19].

An additional case of serendipity is related to the development of another class of ADs, the TCAs. The first TCA, imipramine, was initially tested for the treatment of agitation in patients suffering from psychosis. The drug was ineffective for this purpose, but it was observed to exert antidepressant effects in patients with concomitant depressive symptoms. Subsequent studies clarified the mechanism of action of imipramine, based on the inhibition of the reuptake of serotonin and noradrenaline [20]. TCAs have been used for a long time in the treatment of depression, proving to be very effective, but also showing various side effects determined by their non-specific action on the muscarinic, adrenergic and histaminergic systems, such as drowsiness, sedation, hypotension, dry mouth, constipation and blurred vision, that exert a strong negative impact on adherence to therapy [21].

Based on these first observations, the pharmacological research on ADs moved for the first time in the direction of a “conscious” development of new drugs characterized, in addition to an efficacy profile similar to that of TCAs, also by a better tolerability. This led to the formulation of specific reuptake inhibitors for the serotonergic and noradrenergic systems. Fluoxetine, sertraline, paroxetine, fluvoxamine and citalopram were the first SSRIs to be developed; they act by blocking the serotonin transporter (SERT), while reboxetine is the prototype of SNRIs [20]. Although SSRIs have been formulated on the basis of the first theory on the pathogenesis of depression, i.e., the monoaminergic theory, they still represent the first-line treatment for MDD in clinical practice [22]. 

However, the monoaminergic theory, based on the assumption that the dysregulation of single neurotransmitter systems underlies a complex mental pathology such as MDD, was likely to be an over-simplistic solution. Furthermore, the monoaminergic theory of depression brought a paradox within itself: indeed, the improvement of symptoms in patients treated with ADs is observed after some weeks from the beginning of therapy, whereas experiments conducted on animals showed that the increase in monoamine levels occurred within a few hours after administration [23,24]. The long period between the beginning of the therapy and the manifestation of its effects at the symptomatological level is a relevant problem in clinical practice, also considering that, on the contrary, many of the side effects of ADs become evident within a few hours or days, greatly limiting patients’ compliance [25]. How could such a long latency for the manifestation of clinical effects be explained? This enigma remained unsolved for more than 40 years until, at the beginning of the 1990s, the formulation of a new molecular hypothesis of depression, i.e., the **neurotrophic hypothesis**, through its coupling with the previous monoaminergic theory, found an answer to this apparent contradiction. This new theory was formulated from experimental observations that had revealed how ADs are able to exert an action on neurotrophins or neurotrophic factors [26]. These molecules support the development and proper functioning of neurons and promote resistance and regeneration when necessary, such as in response to insults and stressful stimuli. The first neurotrophin to be described was the neural growth factor (NGF), identified by the Italian scientist Rita Levi Montalcini in 1954 [27,28]. ADs induce an increase in neuronal trophism, resulting in axon growth and an increase in dendritic arborization and dendritic spine density, which are instead reduced in animal models of MDD. This occurs because these drugs stimulate the activity of the genes encoding NGF and other neurotrophic factors, in particular the brain-derived neurotrophic factor (BDNF) and the glial cell-derived neurotrophic factor (GDNF). However, the mechanism is not immediate, but requires a few weeks to take place; this provides a plausible explanation for the time window required for the observation of the effects of ADs on disease symptoms and indicates that the pharmacological efficacy is not strictly linked to an increase in the level of monoamines, but rather to the restoration of a correct neuronal function, determined by the intervention of neurotrophic factors [29,30].

In support of this theory it has also been observed that after months or years the pharmacological treatment is able to restore the volume of different brain areas involved in controlling mood, such as the hippocampus, the prefrontal cortex and the nucleus accumbens. The size of these regions is reduced in the pathology because of neuronal loss; thanks to neurotrophic factors, neurons proliferate again and this causes a trophic recovery in the affected areas [31]. These observations led to the development of a new research field aimed at developing both new molecules with neurotrophic action able to cross the blood-brain barrier to act directly on neurons, and molecules that can modulate the expression of genes encoding neurotrophic factors [32].

The concept of neurotrophism is closely linked to that of neurogenesis, namely the differentiation and proliferation of new neurons; neurogenesis is strongly involved in adaptive responses to harmful stimuli. Neurogenesis is very intense in the prenatal period and during childhood and adolescence, when the brain is being formed, until it gradually decreases during adulthood and old age. In adults, neurogenesis can be stimulated by the so-called “enriched environment”, that is, rich in positive stimuli, as well as by learning, a healthy diet, physical activity and adequate sleep [33,34]. Concerning this last aspect, it should be underlined that melatonin, a molecule produced by the pineal gland or epiphysis in absence of light that regulates the circadian cycle by inducing sleep, causes a proliferation of neural precursor cells which is inhibited, on the contrary, by the lack of sleep [35]. 

### 2.2. Subsequent Hypotheses: The Neurodevelopmental, Glutamatergic, GABAergic, Inflammatory/Immune, and Endocrine Hypotheses

On the basis of the neurotrophic hypothesis, it was later observed that several factors influencing the maturation of neural circuits involved in affective functions and found implicated in MDD are able to control processes related to neurogenesis, such as axonal development and dendritogenesis, in specific brain areas. This suggested that the depressive pathology could be caused by alterations in the neurodevelopment, leading to the formulation of the **neurodevelopmental hypothesis** [36,37]. Indeed, adversity during the early stages of life (defined as early life adversity, ELA), such as abuse and stressful life events in childhood, is strongly linked to the development of anxiety and mood disorders in adulthood, although the precise molecular processes that mediate this relationship have not yet been clarified [38]. Studies conducted on animals subjected to different paradigms of adverse life events, such as maternal separation, have shown that stressful experiences induce changes in the reactivity of the hypothalamic-pituitary-adrenal axis (HPA) [39]. These changes include alterations in the expression of the corticotropin-releasing hormone (CRH) and in the physiological response to the adrenocorticotropic hormone and corticosterone that determine an increased reactivity to stress in the adult animal. At the neurochemical level, changes are observed in CRH levels and in the dopaminergic, noradrenergic and GABAergic transmission; also alterations in the amount of neurotrophic factors, such as BDNF, are present, connecting the neurodevelopmental to the neurotrophic hypothesis [40,41]. Similar changes are observed in MDD patients who experienced ELA [42]. 

The neurodevelopmental hypothesis is also linked to the monoaminergic hypothesis, as serotonin is crucial for the development of the human brain. Dysfunctions in the serotonergic tone that occur during the early stages of life can have a major impact on the differentiation and maturation of different brain pathways, influencing sensitivity to stressful stimuli and more generally the regulation of emotions. In humans, two genetic variants in the SERT are found: the s (short) and the l (long) alleles. It has been observed that carriers of the s allele, which is linked to a lower expression of SERT, are more sensitive to stress-induced amygdala activation and more prone to an anxious temperament. In s allele carriers, alterations in the expression of different types of serotonin receptors in the brain have also been described [43].

One more neurotransmitter which has been involved in the pathogenesis of MDD is glutamate. Although glutamate has not been recognized as a neurotransmitter until the early 1980s, much later than the monoaminergic transmitters, this amino acid is nowadays accepted as the major excitatory neurotransmitter in the central nervous system (CNS). The roots of the **glutamatergic hypothesis** of depression can be traced back to the early 1990s, when early findings showed that antagonists of the glutamatergic receptor N-methyl-D-aspartate (NMDA) exert antidepressant-like effects [44].

Glutamate is functionally involved in almost all brain activities; many brain neurons and synapses are glutamatergic, and adaptation of glutamate synaptic transmission, a process referred to as neuroplasticity, largely mediates both cognition and emotion. In first instance, glutamate has been associated with cognitive processes, through functional and structural changes occurring in glutamatergic neurotransmission. A wealth of studies, conducted in particular in the hippocampus, has shown that activity-dependent changes in the synaptic strength of central glutamatergic synapses mediate learning and memory [45]. However, a similar involvement of glutamatergic transmission in emotional processes has been less firmly established. Even if for many years brain areas have been classified as predominantly implicated in cognitive (e.g., prefrontal cortex) versus affective (e.g., amygdala) functions, it has been later proposed that complex cognitive-emotional behaviours are based on dynamic networks involving different brain areas, none of which specifically cognitive or affective [46]. Interestingly, most connections between and within these brain areas are glutamatergic. Growing evidence indicates that pathophysiological changes involving the glutamatergic system are associated with dysfunction in cognition and mood. Indeed, both biochemical and magnetic resonance spectroscopy studies conducted in MDD patients have reported a dysregulation in the content of glutamate in brain areas involved in these functions. Even if it is difficult to draw any firm conclusion directly from these findings, the fact that the levels of glutamate are abnormal in several brain regions of individuals suffering from mood disorders suggests that dysregulated processes involving glutamatergic neurotransmission are at play [47].

Since astrocytes, a type of glial cells, are key players in the clearance and metabolism of glutamate within the brain, they have increasingly grown in interest. Astrocytes regulate synaptic glutamate concentrations by taking up glutamate through specific transporters and converting it to glutamine which is subsequently exported and taken up by neurons to re-form glutamate [48]. An increase in extracellular glutamate is harmful for neurons, exerting toxic effects depending on an excessive stimulation of post-synaptic glutamatergic receptors (excitotoxicity). This phenomenon is often associated with stress; indeed, chronic stressful stimuli, which are closely linked to the development of mood disorders, have been shown to induce a reduction in the amount of astrocytes in the hippocampus, resulting in glutamate accumulation and leading to structural alterations [49,50]. 

This is consistent with the fact that antagonists of the NMDA glutamatergic receptors exert antidepressant-like effects, by reducing the detrimental effects of an excessive stimulation by glutamate. These processes include complex interactions involving other kinds of glutamate receptors such as the α-amino-3-hydroxy-5-methyl-4-isoxazolepropionic acid (AMPA) receptor and have been shown to also underlie the mechanism of action of ketamine, an anaesthetic drug whose antidepressant properties have been observed in the first years of the new millennium; recently, ketamine has begun to be used as a rapid-acting antidepressant [51].

The γ aminobutyric acid (GABA) is another aminoacid acting as a neurotransmitter; contrary to glutamate, which plays excitatory functions, GABA is recognized as the main inhibitory neurotransmitter in the CNS. Additionally, GABA has been involved in MDD: the **GABAergic hypothesis** of depression states that defects in GABAergic neural inhibition can causally contribute to MDD and, conversely, that the efficacy of antidepressant treatment depends on their ability to restore a correct GABAergic neurotransmission. Consistent with a causative role of stress for MDD, chronic exposure of rodents to stress results in behavioural alterations, which are associated with reduced density and function of GABAergic synapses. Of note, stress-induced defects in GABAergic inhibition are self-perpetuating since they can trigger the release of glutamate, leading to a chronically dysregulated stress response. Conversely, mechanisms that enhance GABAergic inhibition confer resilience to stress. Some of the most compelling evidence indicating that defects in GABA transmission can contribute to stress-induced depressive-like symptoms comes from studies conducted in mutant mice. Mice in which the production of specific subunits of the GABA receptors was blocked or reduced through genetic manipulation have been observed to exhibit depression-like behaviours, as well as physiopathological features which are typical of animal models of MDD. Overall, these findings indicate that alterations in GABA neurotransmission could play a role in the development of MDD by inducing a disruption of the stress response system [52,53].

Another hypothesis, recently formulated, about the pathogenesis of MDD is the **inflammatory/immune hypothesis**. A large amount of evidence indicates the importance of the role played by inflammation and immunity in MDD. In fact, patients suffering from this pathology show all the typical characteristics linked to inflammatory activation, including an increased blood expression of pro-inflammatory cytokines and their respective receptors, as well as of acute-phase molecules and chemokines. Furthermore, increased levels of different genes and proteins related to innate immunity, such as interleukins 1β (IL-1β) and 6 (IL-6), tumor necrosis factor (TNF) and toll-like receptors 3 (TLR3) and 4 (TLR4), have been observed in the brain of patients with MDD [54,55]. Another peripheral inflammatory biomarker linked to depression, indicated by several studies, is the C reactive protein (CRP) [56,57]. Moreover, it has to be noted that the administration of inflammatory factors is able to “artificially” induce depressive symptoms in non-depressed individuals [58,59], while the pharmacological blockade of inflammatory processes has positive effects on depressive symptoms [60,61]. An evolutionary explanation, although speculative, of the association between inflammation and MDD was elaborated trying to answer the following question: why are the genetic variants associated with a greater vulnerability to depression so common in today’s population? What is the evolutionary advantage conferred by these variants? The genetic variants associated with a high inflammatory response and, therefore, also with depression, were probably preserved during the course of evolution because they increased the probability of survival and reproductive success in environments with a high presence of pathogens in which human beings developed. In case of infection by a pathogen, the behavioural correlates linked to inflammation, represented for example by social avoidance and anhedonia, typical symptoms of depression, could have the function of saving energy resources to fight the infection and promote healing [62]. Some authors also suggested that the cognitive and behavioural alterations linked to depression provide an advantage in the ability to thoroughly analyse complex relational problems and find appropriate solutions, thus playing a social function [63].

Closely related to the inflammatory-immune hypothesis is the **endocrine hypothesis**, linked in particular to the activity of the HPA axis which was mentioned earlier in connection with the neurodevelopment hypothesis. A hyperactivity of the HPA axis is an evident clinical symptom in patients with MDD, with increased secretion of CRH and elevated blood levels of glucocorticoids [64,65]. Some pro-inflammatory cytokines, such as IL-1β, induce an activation of the HPA axis, through a mechanism involving nitric oxide (NO), an inflammatory mediator. During exposure to stressful stimuli, IL-1β induces the expression of the enzyme nitric oxide synthase, which produces NO, contributing to the manifestation of depressive symptom [66]. A key role is also played by prolactin and somatostatin, two neurohormones involved in the regulation of emotional processes. In rats subjected to chronic stress, a paradigm used to induce a depressive-like syndrome in the animal, both prolactin and somatostatin levels are altered, while the administration of ADs restores their physiological concentrations [67]. In this context, an issue that deserves a separate discussion concerns the so-called endocrine-disrupting chemicals (EDCs) or endocrine disruptors, organic pollutants whose presence in the environment has been associated with various disorders of the nervous system. EDCs include dioxins, polychlorinated biphenyls, pesticides and plasticizers, such as bisphenol A; these compounds are particularly resistant to degradation and can cross the placenta and the blood-brain barrier due to their lipophilic properties. EDCs show a high affinity for the receptors of different hormones, in particular estrogens and, by binding these receptors, they interfere with normal endocrine processes. Moreover, by crossing the blood-brain barrier, they are able to influence the functions of the CNS, altering in particular the signalling mechanisms mediated by steroid and thyroid hormones, with effects on the proliferation of neural progenitor cells, on the processes of differentiation, synaptogenesis and myelination and on the function of neurotransmitters, including serotonin. Recent epidemiological data indicated that prenatal exposure to EDC can alter cognitive functions and mental development in children; in addition, exposure to these substances during prenatal and childhood development is associated with an increased incidence of depressive disorders in adulthood [68].

### 2.3. Recent Hypotheses: The Role of Fatty Acids and the Gut Microbiota

In recent years, several studies have indicated an involvement of factors related to nutrition and intestinal function in the pathogenesis of MDD. In particular, important roles played by fatty acids and by the gut microbiota have been described. 

**Fatty acids** are essential for proper brain function; it is sufficient to consider that the brain is constituted for more than half of its dry weight by lipids, of which about one third is represented by polyunsaturated fatty acids (PUFA). PUFAs are essential constituents of cellular and in particular neuronal membranes; among these, long-chain omega-3 polyunsaturated fatty acids (eicosapentanoic acid, EPA, and docosahexanoic acid, DHA) are the main responsible for membrane fluidity and influence the activity of neurotransmitters and transporters [69]. The mechanism of action of ADs includes effects on PUFAs of cell membranes [70,71] and it has also been hypothesized that the structure of the membranes influences the efficacy of these drugs [72,73]. PUFAs also act as regulators of inflammation and of the HPA axis activity [74]. In humans, de novo synthesis of PUFAs is not possible; thus, their availability depends on dietary intake, mainly in the form of omega-3 and omega-6 [69]. Starting from the end of the last century, observational studies have highlighted a relationship between the dietary intake of PUFAs and the prevalence of psychiatric disorders, highlighting how the latter were less widespread in geographical regions where a lot of fish, which constitutes one of the main food sources of PUFAs, is consumed [75,76]. With regard to MDD, reduced blood concentrations of omega-3 and an increased ratio between omega-6 and omega-3 were found in affected patients [77], suggesting that alterations in PUFA metabolism may contribute to the pathogenesis of the disease. A reciprocal relationship between fatty acid metabolism and the HPA axis was also highlighted. A high concentration of cortisol, which reflects a hyperactivity of the HPA axis, was indeed observed in MDD patients [65]. Cortisol decreases the production and increases the breakdown of omega-3 chains [78]; in turn, a decreased concentration of omega-3 stimulates the activity of the HPA axis [79]. In addition to being involved in MDD, dysregulations in PUFAs have also been associated with cardiovascular diseases; interestingly, cardiovascular disorders show a high level of comorbidity with depression [80]. On these bases, a common pathogenic mechanism has been hypothesized and a model has been developed that describes cardiovascular disorders and depression as two sides of the same coin [69]. Recently, protective effects of omega-3 intake against MDD have been described [81,82], while PUFA supplementation, in particular with omega-3, has been tested as an adjuvant approach or in addition to pharmacological treatment, with modest but significant results [83]. Considering the excellent tolerability of PUFAs, the relatively low costs and the beneficial effects on concomitant cardiovascular pathologies, their administration has been proposed as an enhancement strategy in addition to the use of ADs [84].

The **gut microbiota** is represented by all the microorganisms which live in symbiosis with the human organism within the most distal tract of the digestive system. Some of them are considered neutral, other beneficial, and others pathogenic for humans [85]. The role played by the microbiota in mediating the functions of the gut-brain axis has only recently become an object of study, and it has been observed that it involves the neuroimmunological and neuroendocrine pathways, in a bidirectional way [86,87]. The intestinal microorganisms, mainly bacteria, influence the nervous system mainly through the release of chemical messengers that can cross the blood-brain barrier and mediate brain functions, also regulating the permeability of the junctions between the cells of the intestinal mucosa, or activating the afferent fibres of the vagus nerve to the CNS. Concerning chemical messengers, many intestinal bacteria can, for example, produce serotonin and its metabolites, as well as dopamine, adrenaline and fatty acids [88]. This complex communication system has different effects on affectivity, motivation and cognitive functions. Alterations of the microbiota are also linked to inflammatory processes; in pathologies and conditions characterized by high levels of inflammation, such as the irritable bowel syndrome, inflammatory bowel disease, diabetes and obesity, a relationship between microbiota composition and depressive symptoms has been highlighted [89,90]. Already in 1998, in the context of the biopsychosocial model, a connection between mental states and gastrointestinal pathologies had been described [91]. More recently, a comparison between the composition of the microbiota of MDD patients and that of unaffected subjects has shown alterations in terms of abundance and diversity of different bacterial species. In general, both the quantity and variety of bacteria are reduced in the presence of the disease [92]. Two possible cause-effect relationships have been hypothesized to explain the link between alterations of the gut microbiota and MDD. The reduction of specific bacterial populations could precede the decrease in neurotransmitter levels in the brain and contribute, with a mechanism not yet clarified, to the pathogenesis of the disease. Alternatively, the depressive state could induce changes in the microbiota, possibly contributing to determining a more severe phenotype. Experimental data indicate a coexistence of both mechanisms: as the microbiota is able to modulate depressive states, so depressive states are able to modulate the microbiota [88]. A decisive demonstration of the fact that modifications of the gut microbiota can induce depressive symptoms derived from two studies in which a microbiota transplant was performed from patients with MDD to germ-free mice, that is, grown from their birth in a sterile environment and, therefore, totally free of microorganisms, or in rats where the microbiota had been destroyed using antibiotics. Following transplantation, depressive and anxious behaviours occurred in animals [93,94]. Moreover, in animal models of depression and/or anxiety the introduction of specific bacterial populations in the intestine was observed to reduce the symptomatology, modifying the excitability of enteric neurons and the expression of GABA receptors in the brain. These effects are no longer observed following vagus nerve resection, suggesting that it is indispensable for the communication between microbiota and the nervous system [95]. Furthermore, the administration of probiotics determines a suppression of HPA axis activity in animals [96] and an increase in the synthesis of tryptophan, a precursor of serotonin [97]. Concerning the effects exerted by the depressive state on the intestinal microbiota, it has been shown that the induction of a depressive-like and anxious syndrome in the animal through surgical removal of the olfactory bulb induces a higher HPA activity which, in turn, affects intestinal motility and the composition of the microbiota [98]. Finally, the possibility of manipulating the microbiota paves the way to interesting therapeutic perspectives based on the use of probiotics, living microorganisms naturally present in some foods and added to others, as antidepressant agents. In this regard, probiotics containing lactobacilli and bifidobacteria were found to significantly improve symptoms in patients with MDD [99,100,101]. A recent review has also shown that the use of probiotics reduces the inflammatory state present in depression [102]. Further clinical trials are needed to confirm these encouraging results and to define which endophenotypes can benefit most from the application of this new treatment approach which, if proven effective, could represent a significant step forward in the treatment of MDD [103].

## 3. Molecular Mechanisms of Pharmacological Treatments for MDD 

The discovery of all the major classes of ADs occurred before a detailed knowledge of their molecular targets and mechanisms of action. Often, these drugs came into common clinical use when little was known about the biological bases of MDD and, as previously described, studies on their action have frequently offered insights into the relationship between behaviour and biological processes taking place in the brain. However, even today, the understanding of the molecular causes of MDD is limited, though multiple hypotheses have been proposed and, despite the improvement in the available therapeutic options, the present pharmacological treatments for this disease are far from being optimal. Standard pharmacological treatments for MDD mostly act on monoaminergic systems, based on its first pathogenic hypothesis, according to which drugs enhancing monoamine transmitter function were observed to be effective agents [20]. Nowadays, this pharmacological armamentarium is quite rich, including various compounds that can be classified into different classes according to their primary mechanism of action, as described afterward. Other drugs currently used are not included in any of these classes, mainly because their mechanism of action is still being discussed. In the following part of this review, we will briefly describe the primary mechanism of action of the major classes of ADs, following their chronological order of development, and some of the available line of evidence showing other mechanisms involved in their therapeutic effects, in line with the above-mentioned hypotheses of MDD.

### 3.1. Monoamine Oxidase Inhibitors (MAOIs)

Inhibitors of the monoamine oxidase A (MAO-A), one of the enzymes responsible for the degradation of monoamines in presynaptic terminals, have been introduced in the 1950s. They result in an increase in monoamine levels in many regions of the CNS and include the active principles moclobemide and toloxatone. The MAO-A isoform preferentially deaminates serotonin and noradrenaline and, to a lesser extent, dopamine; MAOIs indirectly modulate a variety of postsynaptic monoaminergic receptors and exert a wide-ranging monoaminergic action [104]. MAOIs are not frequently used because of the many side effects related to their potent nonspecific activity on a broad number of cholinergic, adrenergic, and histaminergic receptors, such as orthostatic hypotension, dizziness and reflex tachycardia, dry mouth, blurry vision, constipation, urine retention, weight gain and sedation [105]. These drugs have shown neurotrophic and neuroprotective effects, increasing in particular the levels of BDNF and the proliferation of hippocampal progenitor cells in chronically stressed mice [104,106]. 

### 3.2. Tricyclic Antidepressants (TCAs)

TCAs were released to the market in 1959, few years after the discovery of the antidepressant properties of imipramine by the Swiss psychiatrist Roland Kuhn [107]. Their typical chemical structure consists of a three-ringed (“tricyclic”) structure with an attached secondary (e.g., in the case of desipramine, nortriptyline and protriptyline) or tertiary amine (as for amitryptiline, imipramine, trimipramine, clomipramine and doxepin). In the first case, TCAs show a greater ability to block the reuptake of serotonin, while in the second one they tend to exert a more pronounced blockade of noradrenalin reuptake. The combination of different amine structures and differences in chemical composition affect the binding of TCAs to their receptors, involving a multitude of adverse effects observed also with these drugs [108]. Amitriptyline and imipramine combine the blockade of both the serotonin and noradrenalin transporter, especially in limbic regions; desipramine is the strongest noradrenergic agent, and clomipramine is the most selective drug for the SERT. TCAs may also potentiate dopaminergic transmission. Some TCAs also display a significant affinity for specific subtypes of serotonin receptors, which may contribute to their antidepressant effects [105,109]. Subsequently, in line with the evolution of MDD pathogenic hypotheses, various evidence, obtained both in experimental animal models and in humans, has shown that some TCAs are capable of regulating the expression of different genes, including neurotrophic factors, such as BDNF, and genes encoding for key components of the synapses, crucial for synaptic plasticity, inducing functional and structural changes in the CNS [105,110,111]. As well as for the majority of classical ADs, for TCAs evidence was also provided for a modulation of glutamatergic transmission; for example, chronic treatment with imipramine was shown to reduce glutamate-based excitatory neurotransmission [112], whereas chronic treatment with desipramine was shown to significantly reduce the release of glutamate from synaptosomes (isolated synaptic terminals prepared from neurons for experimental purposes) [113] and to block the release of glutamate induced by stress in rats [114]. In addition, TCAs can modulate the expression and function of glutamate receptors [115]. Similarly to other ADs, TCAs were also reported to have immunomodulatory properties which could contribute to their therapeutic effects. Serum or plasma levels of TNF-α and different inflammatory interleukins (i.e, IL-1β, IL-6, IL-18) significantly decreased in MDD patients treated with TCAs, in parallel with lymphocyte activity [116,117].

### 3.3. Selective Serotonin Reuptake Inhibitors (SSRIs)

SSRIs have been introduced in the 1970s and are nowadays among the most widely used ADs in the world, considered as first-line pharmacotherapy for MDD due to their safety, efficacy and tolerability. The efficacy of SSRIs is similar to that of TCAs, but they are surely preferable in terms of lower incidence of side effects, mainly cardiovascular ones; however, they can cause gastrointestinal, cognitive, and sexual adverse effects [118]. The currently used SSRIs are fluoxetine, sertraline, paroxetine, fluvoxamine, citalopram and escitalopram. The primary mechanism of action of these drugs resides in their selective inhibition of SERT at the presynaptic terminal; the resulting increase of serotonin in the synaptic cleft stimulates postsynaptic receptors for a more extended period, thus potentiating serotonergic neurotransmission. Different SSRIs vary in their pharmacologic profile. Although SSRIs can have more or less selective effects on SERT, with citalopram/escitalopram being the most selective, their effects cannot only be ascribed to a SERT-mediated increase in serotonin levels. For example, fluoxetine and other SSRIs are weak antagonists of the serotonin receptors 5-HT2A and 5-HT2C, and fluoxetine is also a modest antagonist of 5-HT3 receptors [105]. Additionally, going back to SERT inhibition, this represents the initiating mechanism, then followed by a plethora of intracellular events that account for the antidepressant efficacy of SSRI, and probably also for the delayed onset in the therapeutic response. Up to now, several intracellular mechanisms have been associated with the antidepressant action of SSRIs, and the same have often also become a tool for verifying, from a biochemical and molecular point of view, the possible antidepressant action of subsequently developed compounds. Among the many, a key role has been attributed to BDNF, which expression levels are increased by all the ADs, both in crucial CNS areas, such as the prefrontal cortex and the hippocampus, and in peripheral tissues such as plasma and serum. BDNF has been shown to be required for antidepressant response, and an increase in its expression levels has been observed after prolonged, but not acute, AD administration, consistent with the time course required for the onset of therapeutic effects [29,110,119,120]. After chronic SSRI treatment, an increase in the expression of other neurotrophic factors, such as the vascular endothelial growth factor (VEGF) and the fibroblast growth factor 2 (FGF2), were reported [29,110,121]. Several reports also indicated that SSRIs could exert anti-inflammatory effects that could contribute to their therapeutic efficacy, although with conflicting results. Different meta-analyses highlighted that SSRIs can decrease the levels of peripheral inflammatory markers (mainly IL-6, TNF-α and IL-10); although some studies provided evidence that this effect was associated with treatment response, other investigations failed to demonstrate this relationship [122,123,124]. Furthermore, a growing body of evidence suggests that different ADs, especially SSRIs, can also affect the gut microbiota; indeed, ADs have been shown to possess antimicrobial activity, and a relationship has been observed between these drugs and the diversity and complexity of the microbiota [125]. In this context, the so-called “pharmacomicrobiomics” is still in its infancy, but deserves efforts in order to characterize the effects of the microbial metabolism on the efficacy of the currently available ADs and to unravel new targets for MDD treatment.

### 3.4. Selective Noradrenaline and Serotonin Reuptake Inhibitors (SNRIs)

Venlafaxine, desvenlafaxine, duloxetine and levomilnacipran belong to this class of ADs. Venlafaxine was the first SNRI approved by the Food and Drug Administration in 1993 for the treatment of MDD, then followed by duloxetine in 2004. These substances, unrelated to both TCAs and SSRIs, block the neuronal reuptake of serotonin, noradrenaline and, to a lesser extent, dopamine. The pharmacological properties of SNRIs are dose-dependent: at low doses, they behave essentially like SSRIs while, at medium doses, an additional inhibition of noradrenaline reuptake occurs; at high to very high doses, they also weakly inhibit the reuptake of dopamine [126]. Concerning the neurotrophic effects of SNRIs, chronic treatment with duloxetine has been reported to produce an upregulation of BDNF, to affect its subcellular redistribution in the rat frontal cortex [127] and to increase BDNF levels in the serum of MDD patients [128]. Chronic duloxetine administration also normalizes the increase of glutamatergic NMDA receptors induced by chronic stress in rats [129]. 

### 3.5. Noradrenaline Reuptake Inhibitors (NARIs)

The first antidepressant of this class to be developed was reboxetine, which was approved for clinical use in the late 1990s. NARIs express their actions mainly through an increase of noradrenaline at the synaptic level, and can also activate different postsynaptic adrenergic receptors; these drugs also induce a marked increase in dopamine levels, probably via inhibition of its reuptake [105]. As other ADs, NARIs have been shown to induce an increase of BDNF in hippocampal neurons and to affect other signalling pathways associated to neurotrophic mechanism [110,130,131,132]. Chronic administration of these drugs, specifically reboxetine, can also impact glutamatergic neurotransmission, decreasing the release of glutamate [113,114] and increasing the expression of specific subunits of both the NMDA and the AMPA receptors in the hippocampus [115,133].

### 3.6. Noradrenergic and Selective Serotonergic Antidepressants (NaSSAs)

This class of ADs includes mirtazapine and mianserine. The antidepressant effects of these drugs are mainly due to their action as antagonists of α2 presynaptic adrenergic receptors that leads to an increased release of both noradrenaline and serotonin. Moreover, they also act as antagonists of the serotonin receptors 5-HT2A, 5-HT2C and 5-HT3, and this mechanism is thought to underlie the positive effects produced by NASSAs on sleep, helping to restore the circadian cycles that are perturbed in depressive states [105,134]. In line with other ADs, a few studies showed that prolonged treatment with NASSAs increases the expression of BDNF in the rat hippocampus and frontal cortex [135,136]. NASSAs were also suggested to reduce neuroinflammation at different levels: indeed, mirtazapine has been shown to reduce the proliferation of T lymphocytes and to inhibit the production of interferon γ, a key inflammatory mediator [137,138].

### 3.7. NMDA Glutamatergic Receptor Blockers and Other Glutamatergic/GABAergic Drugs

It is now recognized that the glutamatergic system is an important mediator of MDD pathophysiology and a common pathway for the therapeutic action of antidepressant agents. As previously outlined, ketamine is a widely used general anaesthetic and a relatively common drug of abuse, whose antidepressant properties have been observed in the early 2000s [139]. In particular, ketamine has rapid and efficacious antidepressant effects at sub-anaesthetic doses, depending on it action as an antagonist of NMDA glutamatergic receptors [140,141]. In 2019, S-ketamine (the S enantiomer of ketamine), available as nasal spray and intravenous injectable solution, has been approved by the Food and Drug Administration for the treatment of severe depression with suicidal ideation [141]. The antidepressant effects of ketamine have yet to be fully clarified, but it is well recognized that it may serve as a prototype for an entirely new class of ADs. Although ketamine is classified as an NMDA receptor antagonist, non-ketamine NMDA receptor antagonists have been observed to fail in producing robust ketamine-like antidepressant effects; thus, the blockade of NMDA receptors should not be the only mechanism responsible for the clinical efficacy of ketamine, which also involves the AMPA receptors. The prefrontal cortex and the hippocampus are crucial for the antidepressant actions of ketamine. A series of preclinical studies have demonstrated that ketamine induces a transient burst of glutamate in these areas, which is probably responsible for the subsequent synaptic remodelling and resetting of the glutamatergic system [120,142]. The glutamate burst also stimulates the release of BDNF. These effects, observed in animal models, are paralleled by the repair of functional connectivity in depressed patients [143]. Finally, a recent study has demonstrated that ketamine can also modify the composition of the gut microbiota [141]. 

Antagonists of the AMPA receptors have also been proposed as antidepressant agents and have shown antidepressant-like properties in animal models of MDD, still involving an increase of BDNF; however, potential toxicological concerns associated with chronic activation of AMPA receptors, e.g., neurotoxicity and seizures, limit the long-term use of these compounds. Finally, a modulation of the glutamatergic function through metabotropic receptors (mGlu) has been considered as an alternative to therapies acting via ionotropic mechanisms involving the NMDA and AMPA receptors. In particular, the mGlu5 subtype has been the focus of research in this field. mGlu5 receptors have been indicated as promising targets in the light of their close relationship with NMDA receptors, involving a reciprocal regulation and a functional interaction. This association makes mGlu5 receptors an attractive target for indirectly modulating NMDA receptor function, and different compounds acting as negative modulators of mGlu5 receptors demonstrated an antidepressant-like activity in animal models [144]. Concerning the GABAergic system, emerging evidence indicates that drugs enhancing the function of specific GABA receptors exert an antidepressant action, thereby supporting the GABAergic deficit hypothesis of depression [52]. 

## 4. Molecular Mechanisms of Non-Pharmacological Treatments for MDD

Most of the available literature concerning the biological mechanisms underlying the effectiveness of non-pharmacological treatments for MDD refers to ECT since, as previously described, this therapy is one of the most well-established for this disease [2]. Investigations have been conducted about the main biological systems known to be involved in MDD, indicating multiple effects of ECT on these processes. 

In relation to the monoaminergic hypothesis, over the past few decades studies conducted in animal models have begun to delineate the impact of electroconvulsive seizures (ECS), an accepted experimental analogue of ECT, on neurotransmission systems commonly implicated in MDD, mainly the serotonergic and the dopaminergic ones [145]. Concerning serotonin, most of the preclinical findings pointed at an enhancement of serotonergic neurotransmission, reflected by the up-regulation of postsynaptic 5-HT1A and 5-HT2A receptors in different brain areas [146,147,148,149]; however, these results are in contrast with human studies showing that ECT can reduce the binding of serotonin to the same receptors [150,151]. Effects of ECS/ECT on the dopaminergic system have also been described, with greater consistency between data from animal and human studies. In particular, recent findings from studies in humans have corroborated many of the earlier findings obtained in rodents and non-human primates, indicating an activation of the mesocorticolimbic dopamine system following ECS/ECT which involves various levels of regulation, such as dopamine release [152,153,154,155,156,157], receptor binding [158,159,160] and dopaminergic neurotransmission [161,162,163,164,165]. This is supported by the improving effects exerted by ECT on symptoms typically related to dopaminergic functions, including motivation, concentration and attention [145]. Results from studies on rodents also suggested that the therapeutic effect of ECS may be mediated by an increased release of noradrenaline, as indicated by a compensatory reduction in adrenoceptor binding involving, in particular, the α2 subtype. This is consistent with the observation of an increased α2-adrenoceptor binding, due in turn to a decreased release of noradrenaline, in post-mortem brains of depressed suicide completers. Notably, the decrease in adrenoceptor binding has been observed to be more pronounced in frontal cortex areas having strong connections with limbic areas involved in the processing of emotions, such as the amygdala [166].

Another biological hypothesis which has been recalled to explain the therapeutic effects of ECT is the neurotrophic one. At the beginning of the new century, first studies indicated that ECS is able to enhance the neurogenesis in rat hippocampus, in terms of new-born neurons; this happens with a dose-response relationship and shows prolonged duration [167]. The same was later confirmed in non-human primates [168] and further evidence indicated that the neurotrophic action of ECS also involves a rise in the number of synapses and in synaptic density [169]. At least in part, these effects could be mediated by an increase of the intracerebral concentrations of the neurotrophins BDNF and VEGF, as highlighted by many studies [170,171,172,173]. The findings described so far, coming from preclinical models, are paralleled by investigations conducted in humans through neuroimaging techniques which described an enlargement of hippocampal volume following ECT [140,174,175]. Moreover, other studies conducted on peripheral tissues (blood serum/plasma) of MDD patients showed an effect of ECT in increasing the levels of both BDNF and VEGF, suggesting that these modifications could reflect brain changes [170,176]. Overall, these findings indicate that neurotrophic effects could be crucial for the therapeutic effectiveness of ECT.

Recently, inflammatory and immune responses have also been reported in relation to ECT. Based on the relative hypothesis of MDD pathogenesis, the rationale for these studies arose from the observation of inflammatory and immune dysfunction in treatment-resistant depression, for which ECT represents one of the most effective therapies. Following an ECT session, an acute inflammatory-immune response has been described to occur immediately, as part of an acute stress reaction. Indeed, several studies conducted on patients’ plasma consistently showed a short-term increase of interleukins 1 and 6, accompanied by elevated cortisol levels. However, at the end of the course of ECT treatment, a long-term fall in the concentration of both interleukin 6 and cortisol has been observed [177]. Moreover, ECT has been reported to rapidly increase the total number of leukocytes, which then decreases a few hours after the end of treatment [178,179]; more specifically, an increase in the number and activity of particular leukocyte subpopulations including granulocytes, monocytes, and natural killer cells, has been highlighted 15/30 min after ECT [178,180]. Taken as a whole, these results indicate that there is an acute immuno-inflammatory response immediately following an ECT session, which seems to be reversed following the end of the treatment course. In relation to the aforementioned neurotrophic effects of ECT, it has been hypothesized that the reduction in inflammation over the long-term could lead to an increase in the levels of neurotrophic factors, such as BDNF, and to structural and functional changes secondary to ECT [177].

Among the mechanisms which have been proposed to be responsible for the antidepressant effect of ECT, attention has focused also on the role of the endocrine system. In addition to the increase of cortisol, previously mentioned, the short-term effects of ECT also include the release of other HPA hormones, such as the adrenocorticotropic hormone and prolactin, into the blood. Since ECT is associated with an increase in the permeability of the blood-brain barrier, the elevated hematic levels of these hormones can be more easily absorbed and distributed in the cerebrospinal fluid (CSF), facilitating their contact with brain cells and structures in the CNS. An enhanced neuroendocrine activity following ECT has been proven also by dynamic function tests assessing the integrity of stimulatory and feedback regulation of this system, such as the dexamethasone suppression test. The heightened stimulation by HPA hormones has been hypothesized to mediate the remission of depressed mood and the restoring of vegetative functions, such as sleep, appetite, and sexuality, produced by ECT [181]. 

rTMS is a non-invasive and easily tolerated method that changes the excitability at the site of stimulation and produces widespread effects at the network level, with therapeutic benefits for a broad range of neurological and psychiatric disorders including MDD [182]. However, although rTMS is largely used for several CNS pathologies, the cellular and molecular substrates that underlie its clinical effects remain poorly understood, and most studies have investigated the molecular mechanisms focusing mainly on the serotonergic, dopaminergic, and neurotrophic systems. Concerning the serotonergic and dopaminergic systems, the genes which have been studied include the serotonin transporter (SLC6A4, and in particular the 5-HTTLPR polymorphism), the serotonergic receptors (HTR1A and HTR2A) and the catechol-O-methyltransferase (COMT), with few and sparse results [183,184,185]. Regarding the neurotrophic system, BDNF was investigated (both concerning its Val66Met polymorphism and serum/plasma protein levels) since it was supposed to mediate the therapeutic benefits of rTMS, but results are contradictory. Two studies have indicated that MDD patients homozygous for the Val allele (Val/Val) show better responses to rTMS [184,186]; however, the data are still limited and based on very small cohorts. Moreover, according to the most recent meta-analysis on this topic, rTMS failed to increase BDNF and few studies were carried out in MDD patients [187].

Concerning CBT, several studies investigated its effects in MDD patients and most have focused on the inflammation and immune systems, showing a reduction of relative biomarkers after the treatment, although robust researches examining this relationship are few, and the heterogeneity between the studies and populations examined is high [188,189,190,191,192,193]. Some investigations were carried out also concerning the therapeutic effects of CBT on neurotrophic factors with sparse results and no clear findings [191,194].

Finally, regarding VNS and IPT, few studies have been performed to date. Consequently, the molecular effects of these two important non-pharmacological treatments for MDD patients remain unknown [195,196,197].

## 5. Conclusions 

Scientific research has produced important results regarding the molecular bases of MDD, going far beyond the initial monoaminergic hypothesis. The different theories developed successively on the basis of experimental evidence focus on biological systems that are very different one from each other and, to date, none of the hypotheses described in this review prevails as a key factor related to the development of depression. Nonetheless, since MDD is a complex and multifactorial disorder, the various biological hypotheses that have been illustrated, rather than representing alternatives to each other, are likely to all contribute to the development of the disease, interacting with mutual reinforcing effects. In addition, environmental factors probably cause disruptions of several biological systems in different ways from individual to individual. This could be one of the reasons why the results of the attempts to identify molecular systems implicated in MDD vary substantially from one study to another, depending on the population examined.

Similarly, the biological systems underlying response to treatment have not yet been clearly and comprehensively defined. Indeed, scientific evidence in this field has indicated the involvement of several systems already associated with MDD itself. Thus, it is still difficult to understand whether these associations are attributable to mechanisms related to the treatments rather than to particular phenotypes of the disease, generally characterized by greater symptom severity, chronicity and comorbidities which, in turn, represent vulnerability factors related to non-response to both pharmacological and non-pharmacological treatments.
